# Clinical significance of the C-reactive protein-to-bilirubin ratio in patients with ulcerative colitis

**DOI:** 10.3389/fmed.2023.1227998

**Published:** 2023-09-25

**Authors:** Xijing Huang, Ya Liu, Zhou Zhou, Yan Pan, Yinghui Zhang, Caiping Gao, Chong He

**Affiliations:** ^1^Department of Gastroenterology, Sichuan Provincial People's Hospital, University of Electronic Science and Technology of China, Chengdu, China; ^2^Clinical Immunology Translational Medicine Key Laboratory of Sichuan Province, Sichuan Provincial People's Hospital, University of Electronic Science and Technology of China, Chengdu, China

**Keywords:** ulcerative colitis, inflammatory bowel disease, C-reactive protein, bilirubin, mucosal healing

## Abstract

**Background:**

Ulcerative colitis (UC) is a chronic relapsing remitting disease of the colon. Appropriate monitoring of the disease status is necessary for patients to adopt optimal therapy and obtain a better prognosis. Finding an ideal non-invasive biomarker, which is suitable for long-term monitoring in clinical settings will bring a significant benefit to the individualized management of patients with UC. The aim of this study is to determine the clinical significance of a novel optimizing serological biomarker by integrating C-reactive protein (CRP) and bilirubin levels in monitoring disease activity.

**Methods:**

A total of 182 patients with UC were retrospectively enrolled. Clinical characteristics and laboratory parameters of the subjects were retrieved from the electronic medical record database of our hospital. The CRP-to-bilirubin ratio (CBR) was computed for clinical activity of UC defined by the partial Mayo score and endoscopic activity by the Mayo endoscopic score (MES).

**Results:**

CBR was significantly elevated in patients with UC than that in healthy controls. Patients with clinically or endoscopically active UC showed evidently higher CBR levels compared to those with inactive disease, even in a subset of patients with normal CRP levels. Receiver operating characteristic (ROC) analysis showed that the area under the curve (AUC) of CBR was higher than that of CRP or bilirubin alone for determining clinical remission and endoscopic mucosal improvement. Furthermore, CBR levels were significantly decreased when patients achieved mucosal improvement compared with when they had active endoscopic inflammation.

**Conclusion:**

CBR could be useful to reflect disease activity in patients with UC.

## Introduction

Ulcerative colitis (UC) is a subtype of inflammatory bowel disease (IBD) characterized by refractory and recurrent mucosal inflammation, leading to complications, colonic surgery, and an increased risk of colorectal cancer. The prevalence of UC is rising globally, particularly in newly industrialized regions like China (1–3). The application of immunomodulators and biologics has shifted the objective of IBD treatment from clinical remission to mucosal remission. Recent studies have emphasized the importance of mucosal healing since failure to mucosal healing is a significant predictor of clinical relapse (4).

Endoscopy is considered the diagnostic gold standard for the assessment of mucosal disease activity in UC. However, since endoscopy is invasive and expensive and patients will need repeated examinations in their lifetimes, the application of endoscopy might be limited in long-term follow-up (5). Fecal calprotectin (FCP) is a reliable non-invasive marker for the assessment of mucosal inflammation in UC (6). However, due to the poor compliance of some patients and the complicated collection/processing of fecal samples, it has not been routinely used in clinical practice in China, especially in underdeveloped areas such as Sichuan Province.

In addition, serological markers are widely used in the clinical management of IBD (7). The most intensively evaluated marker was C-reactive protein (CRP), and it has been frequently used as a reference for novel potential markers (8, 9). Recent evidence demonstrates that combined measurements of CRP and serum albumin (the CRP-to-albumin ratio, CRP/ALB) have shown high value in discriminating active disease in patients with IBD. This serologically optimized marker shows better performance than CRP or albumin alone (10, 11). Like albumin, bilirubin is also a significant marker in serum biochemical testing and routine hospital examinations in clinical practice. Bilirubin is a by-product of aged red blood cells' metabolism within the body. In clinical practice, bilirubin has long been recognized as a detrimental metabolite, and its elevated levels are indicative of bile stasis and liver impairment. Earlier studies have indicated that patients with IBD exhibit lower serum bilirubin levels compared to the healthy control group. Moreover, these levels demonstrate a negative correlation with inflammatory markers, such as the disease activity score (12).

In the current study, we amalgamated assessments of CRP and serum bilirubin levels to engender a novel composite, namely, the CRP-to-bilirubin ratio (CBR), which has not been previously published. Our study enrolled 182 patients with UC, enabling us to evaluate the relationships between CBR and both the clinical and endoscopic activities of UC.

## Materials and methods

### Ethical considerations

The protocol of this study was approved by the Institutional Review Board for Clinical Research of Sichuan Provincial People's Hospital, which was in accordance with the Declaration of Helsinki. Since this was a retrospective study and the data were anonymous, the requirement for informed consent was therefore waived.

### Study population

This is a case-control, retrospective study in which a total of 258 participants were enrolled and categorized into two groups, namely, 182 patients with UC and 76 age- and gender-matched healthy volunteers considered controls. We collected follow-up data by reviewing the medical records of patients who were followed up at our hospital. For the patient group, the inclusion criteria were adult patients (age ≥ 18 years) with a confirmed diagnosis of UC. We enrolled healthy volunteers who underwent routine physical examinations in our hospital during the study. Inclusion criteria: individuals without any known acute or chronic medical conditions, no history of recent infections or inflammatory diseases, and who did not complain of any gastrointestinal symptoms. Exclusion criteria: any evidence of acute or chronic illnesses, including infections, autoimmune diseases, metabolic diseases, or chronic inflammatory conditions; history of recent surgeries or trauma; use of medications or medical conditions known to affect CRP levels; hepatobiliary disease (e.g., hepatitis, cirrhosis, and hepatocholangiolithiasis); hemolytic disease; and use of medications or medical conditions known to affect bilirubin levels. Subjects with smoking, excessive drinking, and other gastrointestinal diseases were also excluded.

UC was diagnosed by clinical findings and radiological examinations, as well as colonoscopic and histopathological confirmation, according to the Consensus on Diagnosis and Treatment of Inflammatory Bowel Disease (2012, Guangzhou) (13). The exclusion criteria for both UC patients and controls were as follows: smoking, excessive drinking, hematopoietic system disease, hepatobiliary disease, coagulation abnormalities, hypertension, diabetes, infections, other systemic autoimmune diseases, other gastrointestinal diseases, and cancers. A Partial Mayo Score system (p-Mayo, range 0–9) based on stool frequency, rectal bleeding, and physician's global assessment was applied to determine the clinical disease severity of UC using the following criteria: inactive, ≤ 2; mild, ≥3 and <5; moderate, ≥5 and <7; and severe, ≥ 7 (14). The assessment of endoscopic activity was conducted by proficient gastroenterologists with over 5 years of experience in endoscopy and prior familiarity with various IBD scoring systems. These gastroenterologists were affiliated with our renowned clinical center, the Department of Gastroenterology at Sichuan Provincial People's Hospital, which specializes in the management of IBD. They possessed extensive knowledge of clinical trials involving endoscopic evaluations. To ensure consistency in the interpretation of endoscopic images and the assignment of scores, all gastroenterologists underwent a comprehensive training session. During this session, they thoroughly reviewed the endoscopic scoring system utilized in our study and received comprehensive guidelines and instructions on the methodology of the scoring system. This training aimed to standardize the evaluation process and promote uniformity in the assessment of endoscopic findings. The location of endoscopic lesions was determined based on the Montreal classification system: E1, proctitis, involvement limited to the rectum; E2, left-sided colitis, involvement limited to a proportion of the colorectum distal to the splenic flexure; and E3, extensive colitis (pancolitis), involvement extends proximally to the splenic flexure. The severity of endoscopic lesions was evaluated by the Mayo Endoscopic Score (MES, 0–3 points). Endoscopic mucosal improvement was defined as MES ≤ 1. Endoscopic mucosal healing was defined as MES = 0 (15, 16).

Patients were suggested to follow with approximately bimonthly visit intervals. Follow-up data on patients with UC were obtained by reviewing their medical records. Serum samples from the participants were collected within 3 days before the endoscopy procedure.

### Statistical analysis

Data manipulation and analysis were conducted using the SPSS version 20.0 software (SPSS Inc., Chicago, IL, United States). The Kolmogorov-Smirnov test was used for significant deviation from normal distribution. Comparison of the variables was performed using the Mann-Whitney *U*-test between two groups except for **Figure 4**, in which a Wilcoxon signed-rank test was performed, or the Kruskal-Wallis test followed by Dunn's multiple comparisons test was performed among the three groups. The difference in gender between subjects was determined by the chi-square test. Receiver operating characteristic (ROC) curves were plotted to calculate the area under the ROC curve. A cutoff value was revealed by ROC curves according to the Youden index. The Youden index was calculated as (sensitivity + specificity – 1). Positive likelihood ratios (LR+) were calculated as sensitivity/(1 – specificity). Negative likelihood ratios (LR–) were calculated as (1 – sensitivity)/specificity. *P* < 0.05 was considered statistically significant.

## Results

### CBR levels were elevated in patients with UC

In the present investigation, a total of 258 participants were recruited, comprising 182 individuals diagnosed with ulcerative colitis (UC), encompassing 84 women and 98 men. Age-matched cohorts of healthy individuals were also included, comprising 34 women and 42 men. Within the UC cohort, 42 participants were diagnosed with proctitis, while 140 exhibited left-sided colitis or extensive colitis. A comprehensive account of the clinical and laboratory characteristics of the enrolled subjects is given in Table 1. We conducted a comparative analysis of CBR levels between UC patients and healthy controls. Remarkably, the CBR levels observed in UC patients (4.13, IQR 1.95–6.40) were significantly elevated in comparison to those detected in the control group (0.19, IQR 0.13–0.23). These findings align with previous investigations conducted by our team and others, which have consistently demonstrated a reduction in serum bilirubin and albumin levels, as well as an elevation in CRP levels, among UC patients when compared to healthy controls (Table 1).

**Table 1:** Clinical and laboratory characteristics of enrolled subjects.

	**UC**	**Healthy controls**	***p*-value**
Number of subjects (*n*)	182	76	
Age (year)	43 (34–51)	40 (33–48)	0.2080^b^
**Gender (** ***n*** **)**			
Female	84	34	0.8913^c^
Male	98	42	
Disease duration (months)	34 (23–47)	-	
**Disease extent (** ***n*** **)** ^a^			
E1	42	-	
E2	64	-	
E3	76	-	
**Treatment (** ***n*** **)**			
5-Aminosalicylic acid	150	-	
Corticosteroids	49	-	
Immunomodulators	47	-	
Biologic agents	20	-	
CRP (mg/L)	22.6 (13.2–31.9)	1.8 (1.5–2.8)	< 0.001^b^
Serum bilirubin (μmol/L)	5.4 (4.4–6.8)	10.4 (7.5–13.5)	< 0.001^b^
Serum albumin (g/L)	36.6 (31.2–43.1)	43.4 (42.1–44.9)	< 0.001^b^
CBR	4.13 (1.95–6.40)	0.19 (0.13–0.23)	< 0.001^b^

Data are presented as the median (interquartile range) when applicable.

UC, ulcerative colitis; CRP, C-reactive protein; CBR, the CRP-to-bilirubin ratio.

a Phenotypes of UC were classified according to the Montreal classification system.

b Mann-Whitney U-test, P < 0.05 was considered statistically significant.

c Chi-square test, P < 0.05 was considered statistically significant.

### Higher CBR levels in clinically active UC

**Figure 1:**
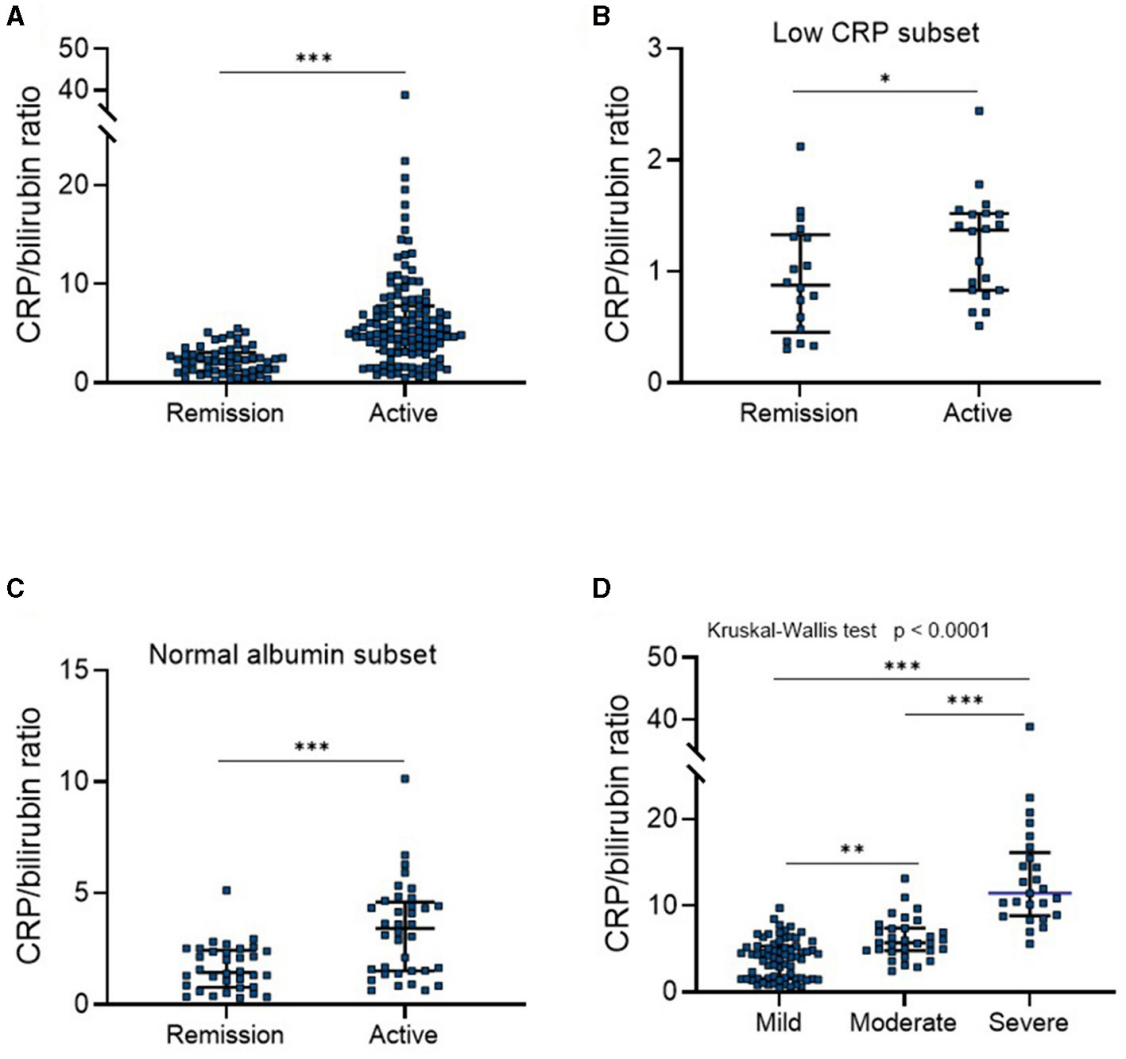
CBR indicates clinical disease activity in UC. All enrolled patients with ulcerative colitis (UC) (*N* = 182) were divided into four groups according to p-Mayo scores: three clinically active and one inactive (remission) groups. **(A)** CBR levels were compared between patients in clinical remission (*N* = 57) and with active UC (*N* = 125). **(B)** The detection of CBR was shown in a subset of patients with low CRP levels (≤ 10 mg/L), consisting of 18 patients in remission and 20 with active disease. **(C)** The detection of CBR was shown in a subset of patients with normal serum albumin levels (40–55 g/L), consisting of 34 patients in remission and 36 with active disease. **(D)** CBR levels were further compared among three groups of active patients. CBR levels of patients with active UC were plotted according to p-Mayo scores (mild, *N* = 69; moderate, *N* = 31; severe, *N* = 25). *P < 0.05, **P < 0.01, ***P < 0.001 determined using **(A–C)** the Mann-Whitney *U*-test and **(D)** the Kruskal-Wallis test followed by Dunn's multiple comparisons test.

Next, we aimed to analyze the relationship between CBR levels and UC activity. To this end, the clinical activity of patients with UC was defined according to the p-Mayo score, and 57 patients were diagnosed with inactive UC (remission). CBR levels were found to be higher in patients with active UC than those in remission (Figure 1A). In this scenario, a cutoff value of 3.93 with a sensitivity of 70.4%, a specificity of 87.7%, an LR (+) of 5.73, and an LR (–) of 0.34 was revealed by ROC analysis. Of note, we found that CBR levels were significantly different between patients with active UC and those in remission, even with low levels of CRP (≤ 10 mg/L, Figure 1B) or normal levels of serum albumin (40–55 g/L, Figure 1C). When patients with UC were categorized into three groups according to disease location by the Montreal classification, CBR was significantly higher in clinically active patients with UC having proctitis, extensive, and left-sided colitis than in those in remission (Supplementary Figure 1A). To further assess the possibility of CBR as a biomarker for disease activity, patients with active UC were divided into three groups, namely, mild, moderate, and severe. As shown in Figure 1D, statistically significant differences in CBR were observed among three groups, and patients with mild UC (a p-Mayo score of 3 or 4) had the lowest levels of CBR, followed by those with moderate UC (a p-Mayo score of 5 or 6). Patients with severe UC (a p-Mayo score ≥ 7) had significantly higher CBR levels than the other two groups. These data demonstrate that CBR indicates the clinical disease severity of UC.

### Correlation of CBR levels with endoscopic mucosal activity in UC

**Figure 2:**
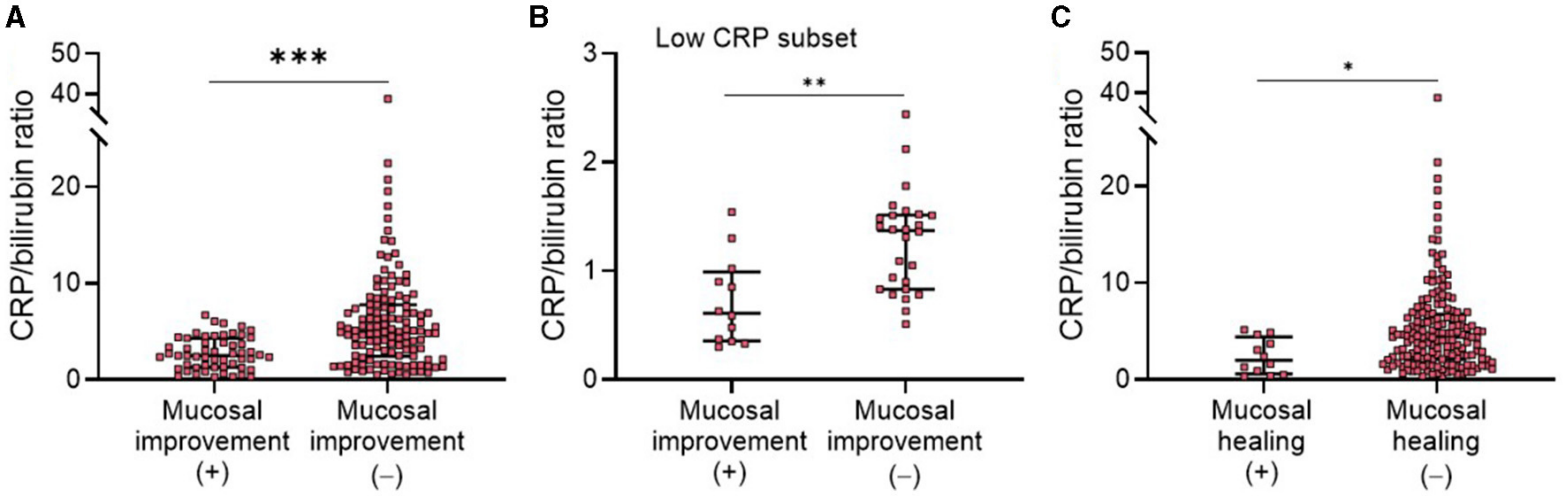
Correlation of NBR with endoscopic mucosal activity in patients with UC. All enrolled patients with UC (*N* = 182) were endoscopically evaluated. According to the Mayo endoscopic score (MES) system, endoscopic mucosal improvement was defined as a MES score of 0 or 1, and mucosal healing was defined as a MES score of 0. CBR levels were compared **(A)** between patients with (*N* = 55) or without (*N* = 127) mucosal improvement or **(B)** in a subset of patients with low CRP levels (≤10 mg/L), consisting of 12 mucosal improvement (+) and 26 mucosal improvement (–) patients. **(C)** CBR levels were compared between patients with (*N* = 12) or without (*N* = 170) mucosal healing. *P < 0.05, **P < 0.01, ***P < 0.001 determined using the Mann-Whitney *U*-test.

Medical treatment for UC is guided by both clinical and endoscopic disease activity, and mucosal healing has been well-known as the key therapeutic target of UC (17, 18). Therefore, we next assessed whether CBR levels were able to reflect endoscopic mucosal activity in patients with UC. Based on endoscopic findings and the Mayo endoscopic score (MES) system, 55 patients with endoscopic mucosal improvement (an MES of ≤ 1) were identified. As shown in Figure 2A, patients who did not achieve endoscopic mucosal improvement had significantly higher CBR levels than those who did, revealing a cutoff value of 3.84 with a sensitivity of 63.8%, a specificity of 74.6%, an LR (+) of 2.51, and an LR (–) of 0.49. Additionally, similar results were observed in patients with UC showing normal CRP levels (Figure 2B). When patients with UC were categorized into three groups according to disease location by the Montreal classification, CBR was significantly higher in patients without mucosal improvement who had proctitis and extensive colitis than in those with mucosal improvement (Supplementary Figure 1B). Additionally, we assessed the value of CBR in the detection of mucosal healing in UC and found that CBR in patients who achieved mucosal healing (MES = 0) was significantly lower than that in those without MH (MES = 1/2/3) (Figure 2C), revealing a cutoff value of 3.09 with a sensitivity of 62.35%, a specificity of 66.67%, an LR (+) of 1.87, and an LR (–) of 0.56. These data suggest an association of CBR with mucosal activity in patients with UC.

### Efficacy of CBR for evaluation of disease activity in UC

**Figure 3:**
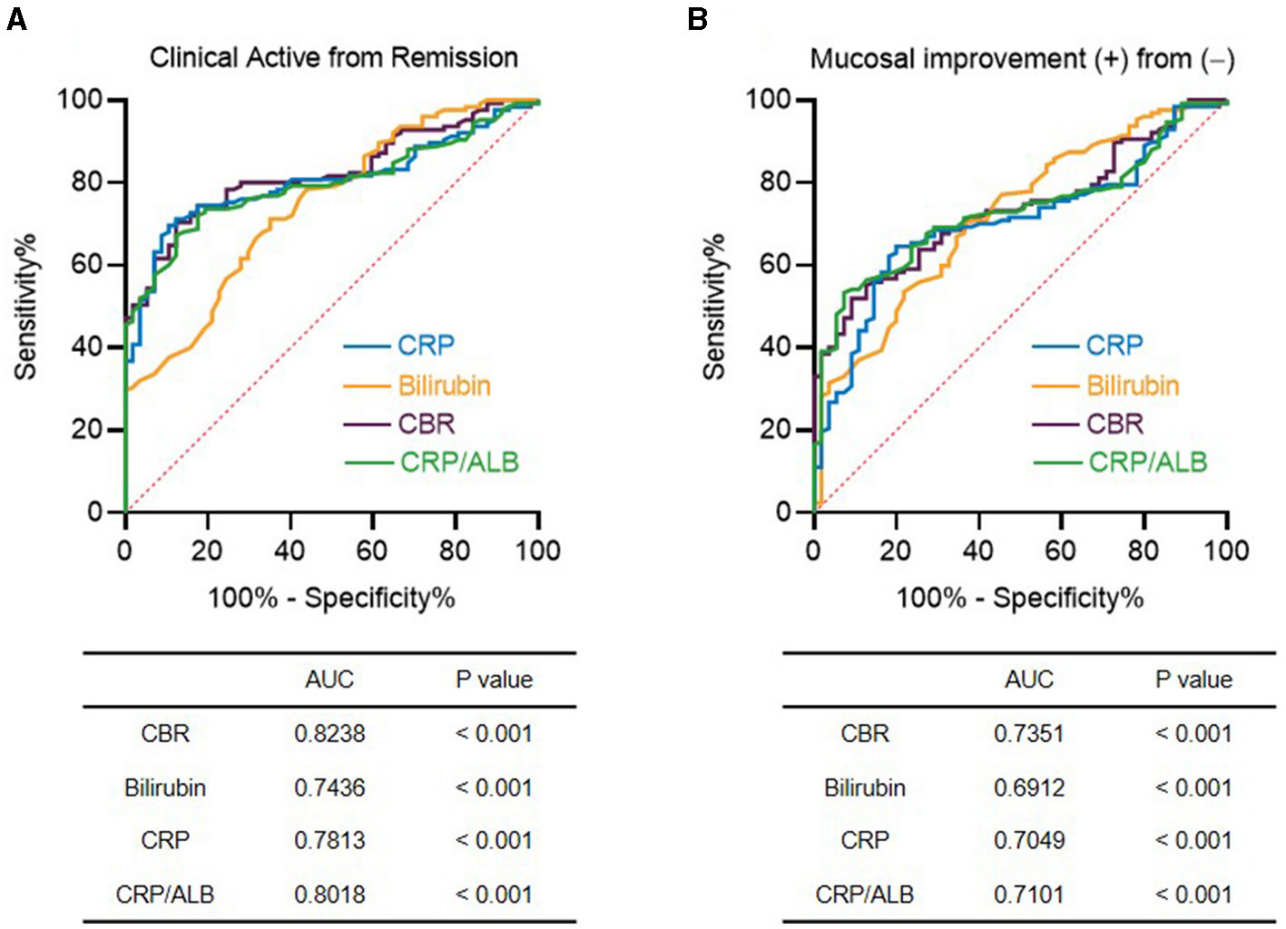
Efficacy of CBR as a serological marker for detecting disease activity in patients with UC. The receiver operating curves (ROCs) are shown for CBR, CRP, bilirubin, and the CRP-to-albumin ratio (CRP/ALB) in determining **(A)** clinical remission and **(B)** endoscopic mucosal improvement in patients with UC. The values of the area under the ROC curve (AUC) are also shown. *P* < 0.05 was considered significant.

According to our findings above, we showed that CBR might be useful to reflect clinical and endoscopic activity in patients with UC. We next investigated its diagnostic accuracy in detecting disease activity by ROC curve and AUC analysis. First, we found that CBR, as an optimizing index, exhibited a higher AUC than CRP or bilirubin alone for the determination of clinical remission by p-Mayo score (Figure 3A) and endoscopic mucosal improvement by MES (Figure 3B). Moreover, the CRP-to-albumin ratio (CRP/ALB) was suggested to be an effective serological optimizing marker in IBD (11, 19, 20). We compared CBR and CRP/ALB in regard to their diagnostic accuracy in detecting disease activity. For clinical remission and endoscopic MH, CBR showed a higher AUC than CRP/ALB (Figures 3A, B). Collectively, these findings strengthen our evidence that CBR is an effective tool to reflect clinical remission and mucosal improvement in UC.

### CBR reflects endoscopic disease activity during the clinical course of the UC

**Figure 4:**
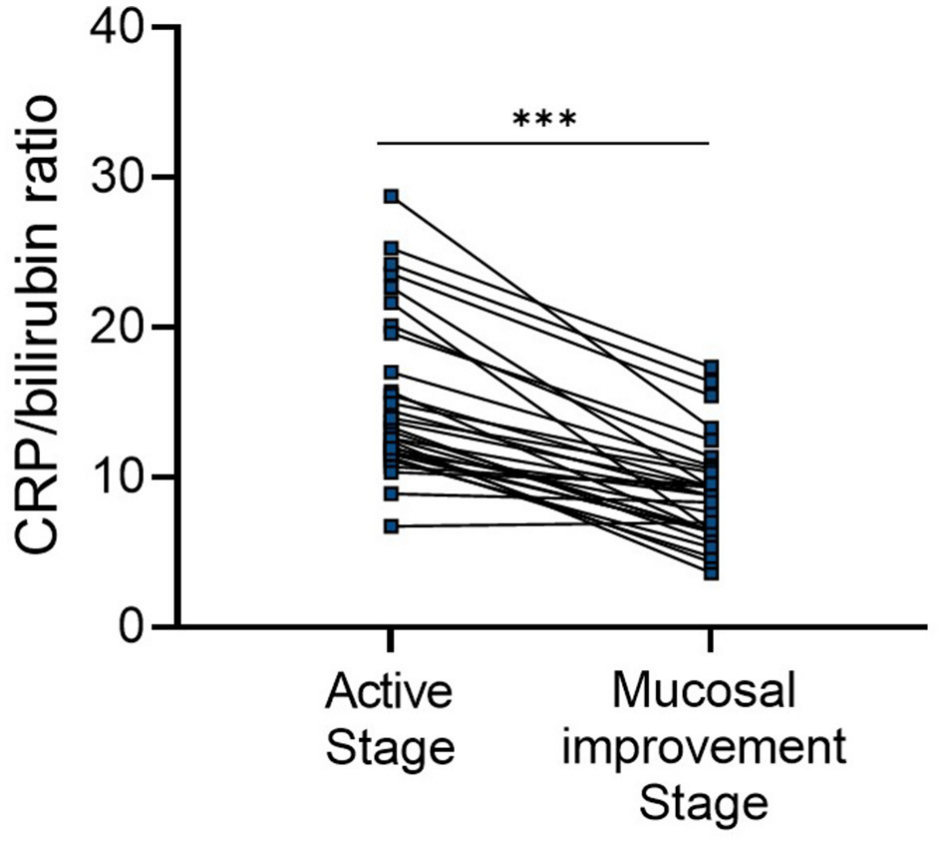
CBR reflects endoscopic disease activity during the clinical course of UC. Patients with endoscopically active UC (a MES score of 2 or 3) at diagnosis were retrospectively analyzed. Endoscopic mucosal improvement (a MES score of 0 or 1) was established in 31 patients after a median period of 26 months (interquartile range: 13–36 months). CBR levels were evaluated at these two time points. ***P < 0.001 was determined using the Wilcoxon signed-rank test.

We next examined the value of CBR as a biomarker to reflect endoscopic disease activity in patients with UC by monitoring changes in CBR levels during the course of the disease. A subset of patients with active mucosal inflammation (an MES score of 2 or 3) at diagnosis and followed up at our hospital were retrospectively analyzed. By reviewing their medical records, we found that during a median period of 26 months (interquartile range: 13–36 months), 31 patients achieved endoscopic mucosal improvement (an MES score of 0 or 1). CBR was measured both when they had endoscopic active inflammation and when mucosal improvement was established. As shown in Figure 4, mucosal improvement achievement was accompanied by greatly decreased CBR levels, suggesting that CBR levels can reflect endoscopic disease activity in UC.

## Discussion

In the current study, we demonstrated that a novel index, CBR, was significantly increased in patients with UC compared to healthy controls. Active UC patients (both clinically and endoscopically) had significantly higher CBR levels compared to those with inactive disease, even in patients showing normal CRP levels. We also found that CBR could reflect endoscopic mucosal activity during the course of UC.

It is necessary to timely assess the disease activity of UC so that patients can adopt optimal therapy and obtain a better prognosis (21). In this study, one major finding was that measurement of CBR gave a good indication of the endoscopic disease activity in patients with UC. Achievement of mucosal healing has been considered to show better prognostic values in the disease outcome than clinical scores (4, 22). In patients with UC, mucosal healing is associated with a lower risk of clinical relapse, hospitalization, need for immunosuppression, and colectomy (23). To evaluate mucosal healing, endoscopy is mandatory. Since UC is relapsing and remitting, frequent endoscopic examinations are occasionally required, especially after starting new treatments. Periodic endoscopic monitoring is also recommended to avoid persistent chronic mucosal inflammation that can lead to colitis-associated neoplasia. On the other hand, due to the cost, invasiveness, and potential risk of disease exacerbation, attempts have been made to find out non-invasive biomarkers (fecal-, serum-, and urine-based), which are ready to surrogate endoscopy with an equally accurate result.

Although there is a wide range of non-invasive markers reported regarding IBD, fecal calprotectin (FCP) remains the most powerful biomarker for mucosal healing (24, 25). However, fecal samples have inherent associated problems such as limited quantitative capability and cumbersome sampling, which are not significant concerns for serum samples. Using serological biomarkers to monitor mucosal healing will avoid repeated endoscopic procedures in patients. Additionally, serological biomarkers are non-invasive, readily available, and not easily contaminated. Among them, CRP is the most widely used (26). CRP is usually induced by acute inflammation and secreted by hepatocytes. Although CRP is widely used in a variety of diseases, it has long been utilized in IBD assessment because its detection is simple and rapid. Due to the non-specificity, recent studies have been carried out to optimize the application of CRP in IBD, such as CRP/ALB, which is a newly-identified optimizing index integrating CRP and albumin and has been reported to be related to the disease activity in CD and UC (10, 11, 19, 20, 27–29). Similar to albumin, low levels of serum bilirubin are also found in patients with IBD (30, 31), so we are encouraged to question whether CBR could be better applied in the evaluation of IBD than CRP or bilirubin alone. Here, we found that CBR was significantly elevated when patients had clinically active UC compared to those in remission. Furthermore, CBR levels were related to the achievement of endoscopic mucosal healing. Importantly, by ROC analysis, CBR showed better performance in reflecting clinical and endoscopic disease activity than CRP or bilirubin alone. Even when compared with CRP/ALB, CBR seemed more appropriate in these two scenarios.

Bilirubin, a by-product of heme degradation, is synthesized through a series of enzymatic reactions involving heme oxygenase and biliverdin reductase. Despite its cytotoxic nature at high concentrations, several clinical studies have revealed an intriguing inverse relationship between bilirubin levels and the risk of IBD. Individuals with Gilbert's polymorphism, characterized by elevated unconjugated bilirubin in the bloodstream, exhibit a reduced susceptibility to Crohn's disease (CD) (31, 32). UC patients have significantly lower serum levels of bilirubin than healthy controls. Bilirubin concentrations are negatively correlated with disease severity and inflammatory marker levels in UC patients (12). In our current study, we found that even when CRP levels were low, serum levels of bilirubin decreased in patients with active UC compared to those in remission (Supplementary Figure 2). Emerging evidence emphasizes the potent antioxidant properties of bilirubin, which are crucial for counteracting lipid peroxidation through scavenging reactive oxygen species (ROS) and inhibiting NADPH oxidase activity (33). The underlying reasons for diminished bilirubin levels in IBD remain unclear, but elevated oxidative stress and heightened production of ROS have been identified in IBD patients (34, 35). These factors may contribute to the increased utilization of endogenous antioxidants, including bilirubin. Notably, studies conducted on animal models have demonstrated that bilirubin exerts a protective effect against colitis by inhibiting colonic leukocyte infiltration and restoring the delicate balance between Th17 and regulatory T cells (Treg).

CBR has its own unique advantages. First, compared with fecal and endoscopic examinations, CRP and bilirubin levels are easily measurable by an inexpensive and noninvasive blood test, and CBR can be quickly computed, making it more suitable for long-term monitoring in clinical settings. Second, besides the fact that CRP itself is an inflammatory indicator of UC, bilirubin has been considered a powerful antioxidant in oxidative stress/inflammation-associated disorders. In UC, decreased bilirubin levels have been reported. Thus, CBR integrates the effects of two inversely related inflammatory pathways, and this biomarker could be more useful to reflect both clinical and endoscopic disease activity in UC than CRP or bilirubin alone, which is confirmed by our results.

Several limitations in the current study warrant consideration. First, it is important to acknowledge that this study was conducted at a single center, and the sample size was relatively small. To establish the clinical significance of CBR, larger sample sizes and multi-center studies are essential for validation. Second, it is worth noting that histological activity was not assessed in this cohort. Future investigations focusing on the correlation between CBR and histological remission are imperative to provide a more comprehensive understanding. Third, the evaluation of endoscopic disease activity solely relied on the MES scoring system. The utility of CBR levels should be further validated using alternative scoring systems such as the UCEIS or the DUBLIN. Expanding the assessment to incorporate additional scoring systems will enhance the reliability and applicability of CBR in evaluating disease activity. Finally, an important limitation of this study is the absence of an investigation into the association between CBR and FCP. Considering the significance of FCP measurements in assessing intestinal inflammation, it is crucial to explore the comparative value of CBR and FCP levels in patients with UC. Future studies should prioritize the inclusion of both biomarkers to obtain a more comprehensive understanding of CBR and ascertain its utility in UC management.

In conclusion, our study highlights the clinical association of CBR with disease activity in patients with UC, indicating the potential of utilizing CBR in the future clinical management of UC.

## Data availability statement

The original contributions presented in the study are included in the article/Supplementary material, further inquiries can be directed to the corresponding author.

## Ethics statement

The studies involving human participants were reviewed and approved by the Institutional Review Board for Clinical Research of Sichuan Provincial People's Hospital (Nos. 201685 and 2020204). The patients/participants provided their written informed consent to participate in this study.

## Author contributions

Conceptualization and writing—review and editing: CG and CH. Funding acquisition and writing—original draft: CH. Methodology: XH, YL, and CH. Validation: XH, ZZ, and YP. Writing—original draft and methodology: YZ. All authors have read and agreed to the published version of the manuscript.
