# Supplementary material for: Clinical significance of the C-reactive protein-to-bilirubin ratio in patients with ulcerative colitis
Source: Front Med (Lausanne). 2023 Sep 25;10:1227998. doi: 10.3389/fmed.2023.1227998 (PMC10560853; doi:10.3389/fmed.2023.1227998)
Supplement: Supplementary file 1 [file Data_Sheet_1.docx]

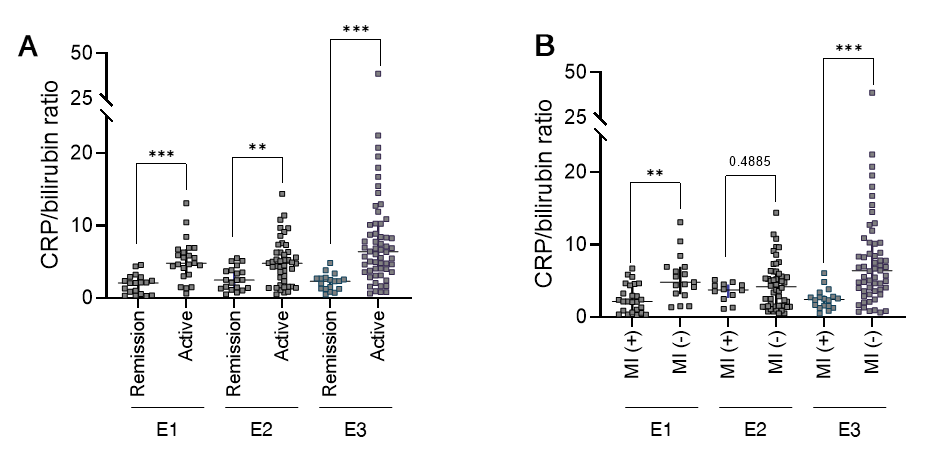


**Supplementary Figure 1.** CBR levels in patients with UC categorised according to (A) clinical activity, (B) mucosal improvement and disease location are shown. ***P* < 0.01, ****P* < 0.001, determined using Mann-Whitney test.


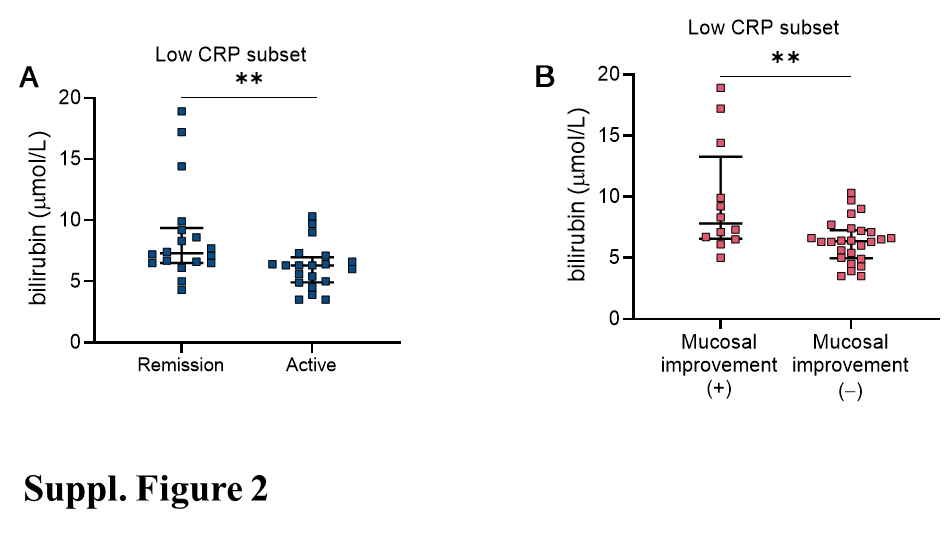


**Supplementary Figure 2.** The serum levels of bilirubin in a subset of UC patients with low CRP levels. The detection of bilirubin was shown in a subset of patients with low CRP levels. Bilirubin levels were compared between a subset of patients (A) in clinical remission and with active UC; (B) with or without mucosal improvement. ***P* < 0.01 determined using Mann-Whitney test.
